# A robust optimization model for allocation-routing problems under uncertain conditions

**DOI:** 10.1371/journal.pone.0322483

**Published:** 2025-05-16

**Authors:** Tingting Zhang, Yanqiu Liu

**Affiliations:** 1 School of Management, Shenyang University of Technology, Shenyang, China; University of Vienna, AUSTRIA

## Abstract

Post-earthquake emergency logistics faces significant challenges such as limited resources, uncertain casualty numbers, and time constraints. Developing a scientific and efficient rescue plan is crucial. One of the key issues is integrating facility location and casualty allocation in emergency medical services, an area rarely explored in existing research. This study proposes a robust optimization model to optimize the location of medical facilities and the transfer of casualties within a three-level rescue chain consisting of disaster areas, temporary hospitals, and general hospitals. The model accounts for limited medical resources, casualty classification, and uncertainty in casualty numbers. The Trauma Index Score (TIS) method is used to classify casualties into two groups, and the dynamic changes in their injuries after treatment at temporary hospitals are considered. The objective is to minimize the total TIS of all casualties. A robust optimization approach is applied to derive the corresponding robust model, and its validity is verified through case studies based on the Lushan earthquake. The findings show that data variability and the uncertainty budget play a critical role in determining hospital locations and casualty transportation plans. Temporary hospital capacity significantly influences the objective function more than general hospitals. As the problem size increases, the robust optimization model performs better than the deterministic model. Furthermore, uncertainty in casualty numbers has a more significant impact on serious casualties than moderate casualties. To enhance the model’s applicability, it is extended into a two-stage dynamic location-allocation model to better address the complexity of post-disaster scenarios.

## 1 Introduction

Earthquakes are among the most devastating natural disasters faced by humanity, occurring frequently and causing significant disruption to economic and social development. They directly threaten human life and hinder the advancement of socioeconomic systems. Throughout history, major earthquakes have had profound impacts. For example, on October 8, 2005, a magnitude 7.6 earthquake struck the South Asian subcontinent, resulting in over 70,000 deaths and more than 60,000 injuries. In April 2010, a magnitude 7.1 earthquake occurred in Yushu, Qinghai Province, China, with a rupture zone about 51 kilometers long, leading to 2,698 deaths and 270 missing individuals, inflicting significant suffering on the local population. Similarly, the Great East Japan Earthquake in 2011 claimed 14,063 lives and caused economic losses amounting to 235 billion. More recently, on December 18, 2023, a magnitude 6.2 earthquake occurred in Jishishan County, Gansu Province, China, at a depth of 10 kilometers, leading to 127 deaths and 734 injuries. These disasters have caused severe social and economic impacts and brought many challenges to post-disaster relief, including complex issues such as facility location, casualty transportation, and resource allocation. Emergency logistics plays a key role in addressing these challenges by rapidly delivering medical equipment, medications, food, and other supplies to the disaster area. As an important part of emergency logistics, emergency medical rescue provides important support for treating casualties.

Effective emergency medical rescue after a disaster depends on providing timely treatment to casualties, which can significantly reduce mortality rates. The "on-site treatment" model is one of the key approaches to improving rescue efficiency [1, 2]. Establishing temporary hospitals at health stations or clinics near the disaster area allows for rapid initial treatment and minimizes the risk of secondary injuries that could occur during long-distance transport. Subsequently, casualties can be transferred to general hospitals for advanced medical care. However, earthquakes often destroy communication infrastructure, block transportation, create complex disaster-area environments, and delay information flow, which makes it difficult to obtain accurate real-time casualty data. In such scenarios, decision-makers must identify optimal locations for temporary and general hospitals and develop effective strategies for casualty transfer and allocation with uncertainty in casualty numbers. Additionally, given limited resources, classifying casualties based on injury severity is essential to ensure efficient transfers and targeted treatment. Various methods, such as the Trauma Index Score (TIS), Trauma Score (TS), Revised Trauma Score (RTS), CRAMS Scale, and Injury Severity Score (ISS), have been extensively used for triage in mass casualty incidents [3–7]. The TIS, introduced by Kirkpatrick *et al*. [6], and the ISS, proposed by Baker *et al*. [7], are among the most commonly employed methods. These methods facilitate the systematic classification and treatment of casualties, allowing for a more targeted and efficient deployment of medical resources. For example, Gong *et al*. [8] utilized the TIS to classify casualties into three categories and optimized the transfer and dispatch plans by considering casualty conditions and road congestion to minimize the total TIS. Wang *et al*. [9] applied the ISS to monitor injury progression among mildly and severely injured casualties and proposed a mathematical model to reduce the total ISS. Similar to the work of Gong *et al*. [8], this study employs the TIS for casualty triage, classifies casualties into moderate and serious categories, and accounts for the deterioration of casualties over time during transportation. The TIS is used to quantitatively evaluate this deterioration.

Stochastic programming methods are commonly used to handle uncertainties in emergency logistics, but they have significant limitations in real-world disaster scenarios. Accurately determining the probability distribution of casualties in advance is often infeasible, and enumerating all possible uncertain scenarios is impractical, which makes stochastic programming less effective or even inapplicable in dealing with real-world uncertainties. In contrast, robust optimization provides a practical alternative by restricting uncertain parameters to specific intervals, eliminating the need for precise probability distributions. In this study, we adopt the robust optimization framework proposed by Bertsimas and Sim [10] to develop an emergency logistics model that defines uncertainty as interval data. The model integrates facility location and casualty transportation under uncertain casualty numbers to minimize the overall TIS across all casualties. We derive the robust counterpart model of the proposed stochastic model using the robust optimization method. The model’s effectiveness is validated using data from the Lushan earthquake, demonstrating its capability to handle uncertainty and produce robust optimal solutions. Furthermore, the impacts of robust optimization parameters (such as data variation and uncertainty budget) and the number of tools (vehicles and helicopters) on the objective value are analyzed.

The main contributions of this paper are as follows:

(1) The paper develops a robust optimization model to address casualty number uncertainty by integrating emergency medical facility location with casualty transportation under uncertain conditions. Compared to the deterministic model, the proposed robust optimization model is more practical and improves rescue efficiency.
(2) We use the TIS method to classify casualties into two categories and account for changes in their injuries after treatment at temporary hospitals.
(3) The paper analyzes the impact of robust optimization parameters (data variability and uncertainty budget) and casualty number uncertainty on the robust model solution while examining how hospital capacity influences the results.
(4) An extended two-stage dynamic location assignment model is proposed to address the uncertainty of casualties and improve its applicability. It enables decision-makers to adjust plans based on real-time data and provides valuable guidance for real-world rescue missions.

The structure of this paper is organized as follows: Section 2 reviews the related literature and highlights the key contributions of this study. Section 3 defines the problem and develops the mathematical model. Section 4 provides a detailed formulation of the robust counterpart of the proposed optimization model. Section 5 demonstrates the model’s application through case studies based on the Lushan earthquake and presents the computational results. Section 6 explores an extension of the proposed model. Finally, Section 7 concludes the study and outlines directions for future research.

## 2 Literature review

At present, a large number of scholars have studied the problems related to disaster relief logistics. As the robust optimization model proposed in this paper focuses on integrating emergency facility location and casualty transportation, the relevant literature is reviewed from four perspectives. In Section 2.1, we review studies on emergency facility location. In Section [subsection2]2.2, we examine the literature related to casualty transportation. Section 2.3 discusses research on triage treatment for casualties, while Section 2.4 explores disaster relief logistics problems addressed using robust optimization.

### 2.1 Facility location problem

The location of emergency facilities is a crucial component of emergency logistics systems and has been a focus of scholarly research since the 1950s [11–14]. Research in this field has primarily centered on models based on demand determination. For example, Gu *et al*. [15] developed a model to locate temporary emergency medical service stations, considering budget constraints and injury severity to maximize the number of people receiving medical care. Moeini *et al*. [16] proposed a dynamic ambulance location model for urban emergency medical systems, considering service reliability and costs, and solved it using the CPLEX solver. Liu *et al*. [17] investigated the location of urban emergency medical stations, focusing on service reliability and employing a genetic algorithm for optimization. Chen and Yu [18] integrated induced demand and traffic networks into an integer programming model for post-disaster temporary emergency medical facility locations, solving it with a greedy algorithm. Ahmadim *et al*. [19] proposed a multi-site location-routing model accounting for network disruption and standard rescue times, extending it into a two-stage stochastic programming framework with random travel times to identify optimal distribution center locations.

In recent years, facility location under uncertainty has attracted growing attention from scholars [10, 20–22]. Hu *et al*. [23] studied a multi-period scenario addressing material demand and transportation time uncertainties using the mean-CVaR method in multi-level emergency logistics networks. Noyan *et al*. [24] analyzed post-disaster demand and transportation network uncertainties to determine the location and capacity of relief supply distribution centers for last-mile distribution. Alizadeh *et al*. [25] considered uncertainties in casualty numbers, injury severities, and evacuation capacities and developed a scenario-based two-stage robust stochastic optimization model to locate casualty collection points and allocate resources. Fereiduni and Shahanaghi [26] addressed disaster dynamics and infrastructure disruptions, such as bridge failures, using a robust optimization model for facility location and service allocation. Du and Zhou [27] studied a P-center location model with robust optimization, solving it using the Gurobi solver. Liu *et al*. [28] proposed a distributed robust location optimization model for emergency service stations and used an outer approximation algorithm to minimize the expected total costs. Zhang and Jiang [29] developed a robust location optimization model for emergency medical service stations and solved it using the CPLEX solver. Gan [30] addressed uncertainties in material demand and transportation times during significant disasters with a multi-objective robust location model. Xu *et al*. [31] proposed a bi-objective model for emergency logistics facility location under uncertain cost, time, demand, and road conditions and used stochastic programming and robust optimization to address the uncertainties.

### 2.2 Casualty transportation problem

After a mass casualty incident occurs, the number of casualties often increases rapidly within a short period. It is crucial to provide timely assistance to casualties. Several scholars have explored challenges related to casualty transportation and scheduling [32, 33]. For instance, Na and Banerjee [34] classified casualties based on on-site diagnoses and assigned rescue vehicles according to the medical resources required for different types of casualties. They proposed a mixed-integer linear programming model to minimize transportation costs. Sung and Lee [35] considered the availability of medical resources at hospitals and the capacity limitations of rescue vehicles. They reframed the casualty treatment issue as an emergency ambulance dispatch model to maximize the expected survival rate and developed a column generation algorithm to address the problem. Liu *et al*. [36] considered factors such as casualty transportation, health status deterioration, and the equitable distribution of limited medical resources. Utilizing ambulances and helicopters as transport modes aimed to maximize the expected number of survivors while minimizing total operational costs. They developed a bi-objective optimization model to determine the optimal locations for temporary medical service points and allocate medical resources effectively. Caunhye *et al*. [37] focused on casualty transportation in the context of catastrophic radiological events to minimize transportation time. They incorporated casualty classification and medical facility categorization to construct a post-disaster medical facility location-casualty transportation model. Sun *et al*. [1] addressed the time-sensitive deterioration of casualties and proposed a robust optimization model for facility location and casualty transportation under uncertainty. Dean and Nair [38] examined factors such as casualty classification, various vehicles’ rescue capacity, and medical institutions’ resource limitations. They developed a model to optimize casualty transportation to maximize the expected number of survivors. Kursat and Itir [39]proposed a multi-objective stochastic programming model for long-term planning of medical center location, casualty transportation, and medical personnel allocation under uncertainty. Andrés B and Diego [40] focused on large-scale casualty disasters, evaluating victims’ age, injury severity, and the potential for deterioration. They explored the optimization of patient transportation routes and proposed a priority strategy for transporting critical casualties to maximize survival probabilities.

### 2.3 Casualty classification problem

In the immediate aftermath of an earthquake, casualties with varying levels of injury severity emerge in large numbers within a short period, often overwhelming the local medical system’s treatment capacity. Differentiating injury severity and integrating casualty prioritization with transportation planning have become critical challenges. Several studies have addressed the classification and treatment of casualties. For instance, Mills *et al*. [41] explored the relationship between the survival probabilities of different categories of casualties and how these probabilities change over time. They built a fluid model for classifying casualties in massive casualty incidents based on the effectiveness of medical resources and the worsening of injuries and outlined its optimal strategy. Yang *et al*. [42] proposed a two-stage hybrid robust programming model that integrates the scheduling of multiple blood products and evacuation of multiple casualty types under the risks of facility disruptions and uncertainty in casualty numbers to improve rescue efficiency. They classified casualties into two categories under the uncertainty of casualty numbers and used two robust uncertainty sets to handle the uncertain casualty count. Shin and Lee [43] integrated casualty prioritization with hospital location to study resource allocation for emergency medical services in significant casualty incidents. Sacco *et al*. [44] focused on post-disaster casualty management by categorizing casualties based on vital signs, including breathing, consciousness, and pulse rate. They introduced the Sacco Triage Method as a systematic casualty classification and transportation approach. Talarico *et al*. [45] analyzed post-disaster casualty treatment and recommended dividing casualties into groups: those treatable at the scene and those requiring hospitalization. They formulated an ambulance routing optimization model to minimize the weighted waiting time of casualties, which was solved using a large-scale neighborhood search algorithm. Recognizing that injuries may deteriorate over time, Jin *et al*. [46] comprehensively considered the tasks involved in different rescue phases after a disaster, the condition of casualties, and the progression of their injuries. They established a marginal level for post-disaster rescue efforts. They developed a mathematical model for casualty transportation and supply distribution, aiming to maximize the number of survivors exceeding the marginal level. Sun *et al*. [2] classified casualties into two categories based on injury severity and proposed a bi-objective robust optimization model to account for the dynamic worsening of casualties over time. Their objectives were to minimize the total ISS and total operational costs.

### 2.4 Disaster relief logistics based on robust optimization

In emergency rescue systems, researchers have widely adopted robust optimization to address the variability and uncertainty inherent in post-disaster environments [47–49]. Najafi *et al*. [50] proposed a multi-objective, multi-commodity, multi-mode, and multi-period robust optimization model for allocating relief supplies and transporting casualties during the earthquake response phase. Their model accounted for uncertainties in capacity and demand and aimed to minimize the weighted total number of unserved individuals, the weighted total unmet demand, and the total number of rescue vehicles. Similarly, Rahmania *et al*. [51] developed a humanitarian relief supply chain model to mitigate the risk of facility disruptions following earthquakes. Their study considered a two-tier structure comprising public and rescue centers and employed robust optimization to handle uncertainties related to earthquake magnitudes. Liu *et al*. [52] proposed a robust optimization model for major mountainous earthquakes to guide rescue mobilization efforts. The model accounted for transportation time and demand uncertainties, aiming to minimize the total costs associated with transportation plans. Paul and Wang [53] focused on pre-disaster emergency response planning, considering uncertainties in post-disaster facility operations, casualty numbers, and transportation times. They established a robust optimization model aimed at minimizing facility opening costs and psychological deprivation costs caused by delayed delivery of emergency materials, providing solutions for the location and capacity of distribution centers. Keyvanshokooh *et al*. [54] proposed a hybrid robust stochastic optimization model for closed-loop supply chain network optimization, addressing various uncertain parameters. They constructed uncertainty sets and improved the model’s solution speed by reducing scenario strategies. Coco *et al*. [55] focused on a robust optimization-based set covering the location model, developing algorithms to minimize maximum regret values. Zhang *et al*. [56] constructed a four-tier humanitarian logistics network, including disaster sites, temporary medical centers in affected areas, and secondary hospitals. Their model optimized medical facility location, resource allocation, and casualty evacuation strategies. By integrating robust optimization methods, the model effectively addressed critical uncertainties such as casualty numbers and transportation times, enhancing the network’s robustness and the responsiveness of post-disaster medical services. Yin and Xu [57] developed a two-stage robust optimization model to address location-allocation and evacuation planning in disaster scenarios. Their model accounted for uncertainties in relief facility capacity, demand, and transportation link availability, providing a comprehensive approach to disaster response planning. Amani *et al*. [58] used robust optimization methods to handle uncertain parameters such as facility disruption probability, number of casualties, transportation time, and rescue demand. They also proposed a data-driven hybrid scenario-based robust approach to solve the mixed-integer second-order cone programming model.

Table 1 summarizes key related works and their main features to highlight the differences between this study and existing research.

**Table 1:** Comparison of relevant literature with our research.

Article	Facility	Casualty	Consider	Uncertainty	Uncertainty	Objective
	location	transportation	injury	type	approach	function
Gu *et al*. [15]	√	√	√			The number of patients
Noyan *et al*. [24]	√			Demand	Stochastic	ETA
					programming	
Alizadeh *et al*. [25]	√	√	√	Casualty number	Stochastic	Total cost
				transportation	programming	
				capacity		
Liu *et al*. [28]	√			EMS	Distributionally	Total cost
				systems	robust	
Sung *et al*. [35]		√	√			The number of
						expected survivals
Liu *et al*. [36]	√	√	√			The number of survivals
						Total cost
Dean *et al*. [38]		√				The number
						of survivals
Najafi *et al*. [50]		√		Demand	Robust	Total unserved injured people
				suppliers	optimization	Total unsatisfied demands
Liu *et al*. [52]				Demand	Robust	Total cost
				time	optimization	
Paul *et al*. [53]	√			Facility damage	Robust	Total cost
				casualty	optimization	
				time		
Sun *et al*. [59]	√	√		Casualty	Scenario-based	Total cost
				number	robust optimization	
Hu *et al*. [23]	√			Demand	Mean-CVaR	Total time
				time	method	
Zhang *et al*. [56]	√	√	√	Casualty number	Robust	Total cost
				time	optimization	
Wang *et al*. [22]	√			Demand	Fuzzy	Total cost
					programming.	Total time
Yin and Xu [57]	√	√		Supply	Robust optimization	
				Demand	two-stage recoverable	Total cost
					robust optimization	
This paper	√	√	√	Casualty number	Robust optimization	Trauma Index

The gaps between this study and existing research are as follows:

(i) Most studies addressing uncertainty in emergency logistics rely on stochastic programming or fuzzy programming. Although some research uses robust optimization, few focus on post-earthquake emergency rescue logistics. This paper applies robust optimization to address uncertainty in this context.
(ii) Existing research often focuses on demand, transportation time, supply, or cost uncertainties, whereas this study incorporates casualty uncertainty.
(iii) Emergency logistics studies typically aim to minimize cost or time or maximize the survival probability of casualties. In contrast, this paper minimizes the total TIS.
(iv) Most studies examine facility location, casualty transport, and casualty triage as separate issues, and although some studies consider these factors together, few account for the dynamic changes in injury conditions over time and with treatment, which are incorporated into the model in this paper to reflect real-time changes in casualty conditions accurately. Accordingly, we develop both single-stage and two-stage robust optimization models. The former enables simultaneous facility location and casualty transportation decisions, a crucial feature for time-sensitive emergencies. The latter follows the “Here and Now” and “Wait and See” decision structures, which help enhance the model’s flexibility and applicability.

## 3 Problem description

Classifying casualties plays a vital role in improving the efficiency of post-earthquake treatment. The TIS is widely used to evaluate injury severity [6, 60] and incorporates five key parameters: circulation, consciousness, injury location, injury type, and respiration. Each parameter receives a score of 1, 3, 5, or 6 based on severity, and the total TIS, calculated as the sum of these scores, provides a comprehensive measure of injury severity. This approach helps determine the treatment order for casualties in disaster situations promptly and accurately, which optimizes rescue operations.

In this study, casualties are categorized based on their total TIS, with deceased individuals excluded. Those with scores of 17 or higher are classified as serious casualties and require immediate treatment to prevent life-threatening conditions. Casualties with scores between 5 and 16 are classified as moderate, as they are unlikely to face life-threatening issues within the next 2 to 4 hours. Casualties with scores below 5 are considered minor and typically need basic care, such as wound cleaning and bandage application, without posing immediate life risks. As a result, minor casualties are excluded from this analysis.

**Fig 1:**
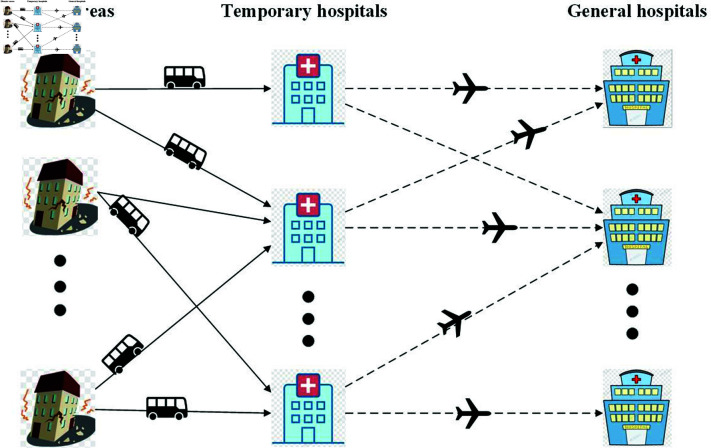
Structure of the proposed emergency logistics network

To enhance survival rates after an earthquake, it is crucial to prioritize the establishment of temporary hospitals near disaster areas. Both moderate and serious casualties are transported to these temporary hospitals by rescue vehicles dispatched from them. After receiving prompt treatment at the temporary hospitals, all casualties are airlifted to general hospitals by helicopters dispatched from the latter. It is important to note that these vehicles return to their original locations after completing their missions. In addition, due to limited resources and medical capabilities, all temporary hospitals have the same capacity for casualties. In contrast, general hospitals have varying capacities, as the heterogeneity of casualties leads to differences in medical resource requirements across injury categories. Based on the above, this study proposes a robust optimization model for disaster response planning to minimize the total TIS for two types of casualties. The model provides solutions for the optimal locations of temporary and general hospitals and transportation planning for casualties. An overview of the emergency rescue network is described in Fig 1.

### 3.1 Problem assumptions

Timely and effective medical intervention is crucial in trauma and disaster response, as it determines the success of rescue efforts [2, 46, 61]. Without prompt professional medical treatment, casualties risk rapid physiological deterioration caused by factors such as blood loss, infection, or shock, leading to a significant increase in their deterioration rate. However, their deterioration rate slows significantly when casualties are transported to temporary hospitals and receive treatments like hemostasis, wound debridement, and blood transfusion. Thus, the speed of transporting casualties to temporary hospitals is critical, as it directly affects their chances of survival. Moreover, serious casualties face higher risks, including massive blood loss, multiple organ failure, and severe infections. In contrast, moderate casualties generally have more stable physiological conditions and a lower risk of deterioration [62, 63].

**Fig 2:**
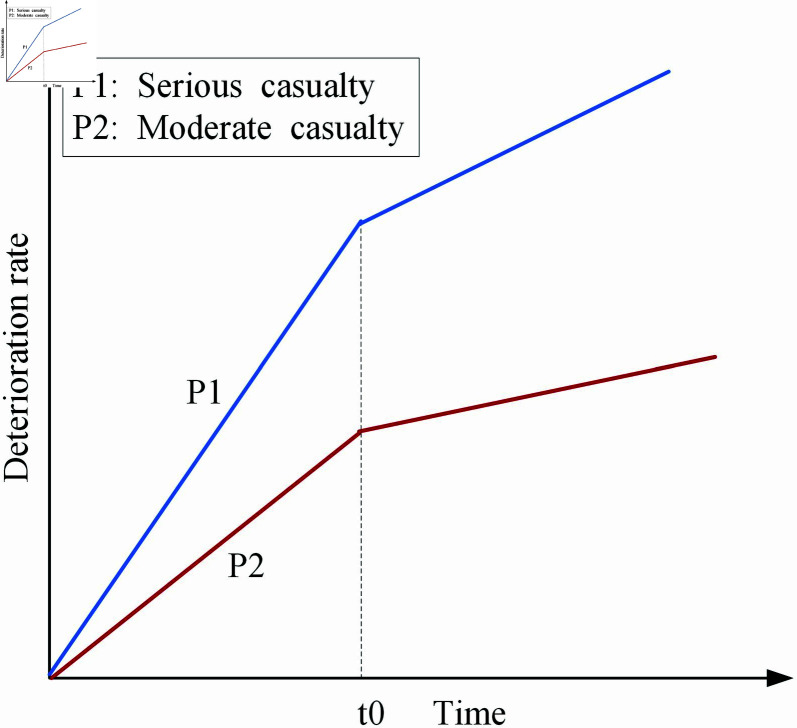
The change in casualty deterioration rate over time.

Based on the above, the trend of the deterioration rate over time is illustrated in Fig 2, where P1 and P2 represent the deterioration rates of serious and moderate casualties, respectively, and *t*0 marks the time when casualties are transported to the temporary hospital. As depicted in the figure, the deterioration rate for serious casualties is consistently higher than that for moderate casualties. Before treatment in the temporary hospital, the deterioration rate of casualties increases rapidly. However, the deterioration rate decreases significantly after receiving treatment in the temporary hospital. Additionally, according to [63], the deterioration rate of casualties’ health conditions decreases to around 20% after receiving emergency treatment.

The main assumptions of the model are as follows: (1) Each temporary hospital can simultaneously serve multiple disaster sites, and the casualties of each disaster area can be assigned to multiple temporary hospitals. (2) Due to the large number of casualties in disaster areas, multiple rescue vehicles can service each disaster area. (3) Each rescue vehicle or helicopter services only to one disaster area or temporary hospital. (4) Rescue vehicles were dispatched from the temporary hospital and helicopters from the general hospital; these tools returned to their original locations after completing their missions. (5) The time for rapid treatment of all casualties at temporary hospitals is not considered. (6) Transporting casualties to general hospitals is considered the end of the rescue operation. These assumptions form the basis for the model development and analysis presented in the paper.

### 3.2 Notation descriptions

In this subsection, we present the mathematical model to be optimized, encompassing its sets, parameters, decision variables, objective functions, and constraints. The notations and definitions used in the proposed model are presented in Table 2 .

**Table 2:** Notation descriptions.

Sets	Descriptions
*I*	Set of disaster areas, i∈I
*J*	Set of candidate temporary hospitals, j∈J
*K*	Set of candidate general hospitals, k∈K
*H*	Set of helicopters, h∈H
*G*	Set of rescue vehicles, g∈G
*S*	Set of casualty types, S={s|s=α,β}, α for serious casualty, β for moderate casualty
**Parameters**	
$\omega_{s}$	Represent the urgency coefficients for serious casualties and moderate casualties, respectively
γ	Total number of vehicles
λ	Total number of helicopters
*e* ^*g*^	The carrying capacity of each emergency rescue vehicle
*e* ^*h*^	The carrying capacity of each helicopter
*t*_*ij*_	Travel time between disaster area i and temporary hospital j by rescue vehicle g (hours)
*t*_*jk*_	Travel time between temporary hospital jand general hospital k by helicopter h (hours)
*q*_*is*_	The number of casualties at disaster area iwith severity level s
*C*	The maximum capacity of temporary hospitals for casualties
*Q*_*ks*_	The maximum capacity of general hospital k for casualties with severity level s
*p*_*s*_	The deterioration rate of casualties with severity level *s*
*p*_*ijs*_	The deterioration rate of casualties with severity level s from disaster area i to temporary hospital j
*p*_*jks*_	The deterioration rate of casualties with severity level s from temporary hospital j to general hospital k
**Decision Variables**	
*U*_*j*_	1 if temporary hospital *j* is selected and 0 otherwise
$V_{k}$	1 if general hospital *k* is selected and 0 otherwise
*X*_*ijg*_	1 if vehicle g travels from disaster area i to temporary hospital j, 0 otherwise
*Y*_*jkh*_	1 if helicopter h travels from temporary hospital j to general hospital k, 0 otherwise
*x*_*ijgs*_	Number of casualties transported by vehicle gfrom disaster area i to temporary hospital j with severity level s
*y*_*jkhs*_	Number of casualties transported by helicopter h from temporary hospital j to general hospital k with severity levels

### 3.3 Mathematical formulation

$$\min F_{1}+F_{2}$$ (1)

$$F_{1}=\sum_{i}\sum_{j}\sum_{g}\sum_{s}p_{ijs}x_{ijgs}\omega_{s}$$ (2)

$$F_{2}=\sum_{j}\sum_{k}\sum_{h}\sum_{s}p_{jks}y_{jkhs}\omega_{s}$$ (3)

$$\sum_{i}\sum_{g}\sum_{s}x_{ijgs}\leq C$$ (4)

$$\sum_{j}\sum_{h}y_{jkhs}\leq Q_{ks}$$ (5)

$$\gamma \geq \sum_{i}\sum_{j}\sum_{g}X_{ijg}$$ (6)

$$\lambda \geq \sum_{j}\sum_{k}\sum_{h}Y_{jkh}$$ (7)

$$\sum_{s}x_{ijgs}\leq X_{ijg}e^{g}$$ (8)

$$\sum_{s}y_{jkhs}\leq Y_{jkh}e^{h}$$ (9)

$$\sum_{i}\sum_{j}X_{ijg}\leq 1$$ (10)

$$\sum_{j}\sum_{k}Y_{jkh}\leq 1$$ (11)

$$X_{ijg}\leq \sum_{s}x_{ijgs}$$ (12)

$$Y_{jkh}\leq \sum_{s}y_{jkhs}$$ (13)

$$\sum_{i}X_{ijg}-U_{j}\leq 0$$ (14)

$$\sum_{j}Y_{jkh}-V_{k}\leq 0$$ (15)

$$\sum_{i}\sum_{g}X_{ijg}-U_{j}\geq 0$$ (16)

$$\sum_{j}\sum_{h}Y_{jkh}-V_{k}\geq 0$$ (17)

$$\sum_{j}\sum_{g}x_{ijgs}=q_{is}$$ (18)

$$\sum_{i}\sum_{g}x_{ijgs}=\sum_{k}\sum_{h}y_{jkhs}$$ (19)

$$p_{ijs}=2t_{ij}p_{s}$$ (20)

$$p_{jks}=2t_{jk}\cdot 0.2p_{s}$$ (21)

$$U_{j},V_{k},X_{ijg},Y_{jkh}\in \{0,1\},\forall j\in J,\forall k\in K,\forall i\in I,\forall g\in G,\forall h\in H$$ (22)

$$x_{ijgs},y_{jkhs}\geq 0,\text{and interger}$$ (23)

Equation (1) is the minimum objective function of the sum of the weighted total TIS of casualties before and after treatment. Equation (2) is the sum of the weighted TIS of moderate and serious casualties before treatment. Equation (3) is the sum of the weighted TIS increment of moderate and serious casualties after treatment. Constraint (4) ensures that the number of casualties brought to the temporary hospital cannot exceed its maximum capacity. Constraint (5) restricts that the number of casualties brought to the General hospital cannot exceed its maximum capacity. Constraints (6) and (7) limit the number of available vehicles and helicopters. Constraints (8) and (9) represent the relationship between the number of casualties transported by each rescue vehicle or helicopter and the carrying capacity of the rescue vehicle or helicopter. Constraints (10) and (11) describe, respectively, that rescue vehicles can only go from one disaster site to the temporary hospital, and helicopters can only go from one temporary hospital to the General hospital. Constraints (12) and (13) indicate that neither the rescue vehicle nor the helicopter can be empty. Constraints (14) and (15) state that rescue vehicles and helicopters can only be dispatched from selected hospitals. Constraints (16) and (17) require the dispatch of at least one rescue vehicle from the selected temporary hospital and at least one helicopter from the selected General hospital. Constraint (18) guarantees that all casualties from each disaster area are transported to temporary hospitals. Constraint (19) means to ensure that casualties from each temporary hospital are transported to the General hospital. Constraint (20) and (21) represent the formula for calculating the deterioration rate of the casualties of type *s*, respectively. Finally, constraints (22) and (23) define all the decision variables.

## 4 Robust model formulation using robust optimization method

In actual rescue operations, the number of casualties is often uncertain due to various factors. To address this, we apply the robust optimization methods proposed by [10, 21] to develop an optimal response plan.

In our proposed model, there is an uncertain variable of the number of casualties in the constraint formula (18). We define the uncertainty in casualty numbers, *q*_*is*_, as an independent, bounded interval $q_{is}\in [{\bar{q}}_{is}-{\hat{q}}_{is},{\bar{q}}_{is}+{\hat{q}}_{is}]$, reflecting the possible fluctuation range. Here, ${\bar{q}}_{is}$ represents the "nominal value" of casualties. ${\hat{q}}_{is}$ represents the maximum deviation from ${\bar{q}}_{is}$. In the model, we introduce a parameter $\Gamma_{i}\in [0,1]$. This parameter represents the uncertainty associated with casualty numbers *q*_*is*_. The parameter $\Gamma_{i}$ adjusts the degree of protection against uncertainty. In other words, $\Gamma_{i}=0$ represents the nominal case without protection against uncertainty, i.e., uncertainty is not considered. $\Gamma_{i}=1$ represents the fully protected case, i.e., the worst case scenario is considered, and when $\Gamma_{i}\in (0,1)$, decision-makers can balance the robustness of constraints with the conservatism of the solution, i.e., partially accounting for uncertainty. The smaller the value of $\Gamma_{i}$, the greater the risk the decision-maker bears. The $\Gamma_{i}$ value depends on the decision-maker’s risk preference.

According to Liu *et al*. [52], the robust optimization method can only deal with stochastic models with constraints of "less than or equal to" inequality, but since the model constraint "$\sum_{j}\sum_{g}x_{ijgs}=q_{is}$" proposed in this paper is not of "less than or equal to" form. Hence, constraint (18) can be rewritten as

$$\sum_{j}\sum_{g}x_{ijgs}\geq q_{is}$$ (24)

With reference to the robust optimization processing method of [64] for the information containing uncertainty on the right, constraint (24) can be converted to:

$$\sum_{j}\sum_{g}x_{ijgs}\geq {\bar{q}}_{is}+\max_{\eta \in Z}\left(\sum_{s}{\hat{q}}_{is}\eta_{is}\right)$$ (25)

Here, $\eta_{is}=\frac{q_{is}-{\bar{q}}_{is}}{{\hat{q}}_{is}}$ is the scaled deviation and $Z=\{\eta ||\eta |\leq 1,\sum_{s}\eta_{is}\leq \Gamma_{i}\}$. We substitute the dual problem of the protection function $\max_{\eta \in Z}\left(\sum_{s}{\hat{q}}_{is}\eta_{is}\right)$ into constraint (25). Thus, when uncertainty affects only the right-hand side value, the robust counterpart of constraint (24) can be rewritten in the following form:

$$\sum_{j}\sum_{g}x_{ijgs}\geq {\bar{q}}_{is}+{\hat{q}}_{is}\Gamma_{i}$$ (26)

To summarize, the robust counterpart of our proposed model is depicted as follows:


$$\min F_{1}+F_{2}$$

s.t. (2)-(17),(19)-(23),(26)

## 5 Case study

To validate the feasibility and effectiveness of our proposed model for addressing the dispatching problem related to rescuing casualties in complex earthquake disaster scenarios, we conducted numerical simulations using actual case data from the 2013 Lushan earthquake in Lushan County, Sichuan Province, China. The model was implemented using MATLAB programming language and utilized CPLEX 12.10 as the solver. Computational solving was conducted in a computing environment with an AMD R7 processor operating at 2.00 GHz and 16 GB of RAM.

### 5.1 Parameter setting

The Lushan earthquake, with a magnitude of 7.0 and an epicenter depth of 13 kilometers, resulted in a significant number of casualties, necessitating urgent rescue operations for the casualties. We primarily referenced historical information, public reports^1^^,^^2^, and the literature [8, 65] on the Lushan earthquake to obtain detailed information on the number of casualties, injury distribution, and the affected areas. Based on these data, we divided the disaster-affected area in Lushan County into nine major disaster areas and selected five nearby health centers as candidates for temporary hospitals. Additionally, considering factors such as proximity to the epicenter and transportation conditions, we identified three large hospitals, which were relatively close to the disaster areas and had better conditions, as candidate general hospitals to meet the post-earthquake medical needs of the casualties and provide emergency medical treatment. I1-I9 represent the disaster areas, J1-J5 represent the candidate temporary hospitals, and K1-K3 represent the candidate general hospitals. For convenience, the following sections will use these symbols to refer to the disaster zones and candidate locations.

Table 3 shows the nominal values of casualties for each disaster area [8], while Table 4 and Table 5 provide the transportation times between disaster areas and candidate temporary hospitals, and between candidate temporary hospitals and candidate general hospitals, respectively. These parameters have been estimated to reflect realistic conditions based on data from relevant literature [2, 66]. Additionally, Table 6 lists the parameter settings for rescue tools. The capacity of each candidate temporary hospital is set at 500 units, and the capacities of each candidate general hospital are detailed in Table 7. During the rescue process, the urgency coefficients for serious and moderate casualties are set at $\omega_{\alpha }=2$ and $\omega_{\beta }=1$, respectively, with their deterioration rates set at $p_{\alpha }=0.8$ and $p_{\beta }=0.2$ [1].

**Table 3:** Nominal number of casualties in disaster areas.

Disaster areas	I1	I2	I3	I4	I5	I6	I7	I8	I9
Serious casualty	59	21	23	29	20	70	12	16	5
Moderate casualty	143	48	71	116	59	153	46	71	23

**Table 4:** Transportation time between disaster areas and candidate temporary hospitals (unit: h).

Disaster areas	Temporary hospitals				
	J1	J2	J3	J4	J5
I1	1.250	0.800	1.200	0.950	0.875
I2	0.575	0.525	0.650	0.525	0.450
I3	1.025	0.825	1.100	0.975	0.775
I4	1.325	0.975	1.375	1.125	1.050
I5	1.475	1.125	1.525	1.300	1.200
I6	1.525	1.175	1.575	1.350	1.250
I7	1.750	1.375	1.675	1.550	1.450
I8	1.725	1.525	1.875	1.825	1.600
I9	2.450	2.250	2.450	2.400	2.325

**Table 5:** Transportation time between candidate temporary hospitals and candidate general hospitals (unit: h).

Temporary hospitals	General hospitals		
	K1	K2	K3
J1	0.57	0.71	0.59
J2	0.63	0.73	0.61
J3	0.57	0.71	0.59
J4	0.60	0.70	0.58
J5	0.58	0.71	0.59

**Table 6:** Parameters about rescue tool.

Rescue tool	Velocity (km/h)	Capacity (units)	Quantity (units)
Rescue helicopters (MI-171)	240	12	280
Rescue vehicles (Dongfeng EQ240)	40	6	150

**Table 7:** Capacity of candidate general hospitals.

General hospitals	Type of casualty	
	Serious casualty	Moderate casualty
K1	250	350
K2	200	300
K3	200	500

### 5.2 Computation results under nominal values

**Fig 3:**
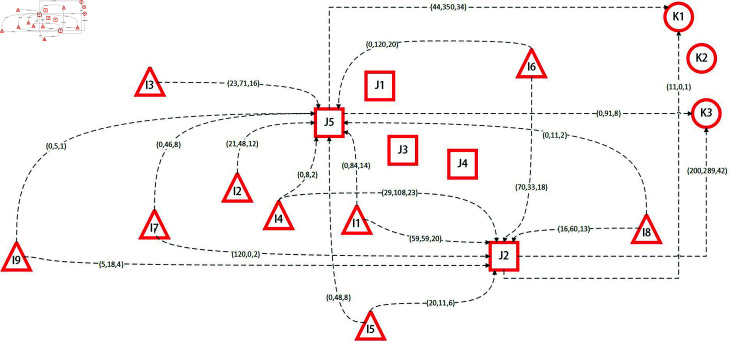
Decision scheme of the deterministic model based on nominal data.

In this subsection, the deterministic model is solved using nominal values without accounting for the uncertainty in casualty numbers. MATLAB and the CPLEX solver are used to optimize transportation routes from each disaster area to candidate hospitals and to select the most suitable temporary and general hospitals for treatment. The model considers transportation time, hospital capacity, and the number of rescue tools to ensure that casualties receive effective treatment in the shortest possible time. The specific transportation routes and hospital locations are illustrated in Fig 3.

Fig 3 illustrates the casualty transport scheduling derived from deterministic data in the deterministic model. Temporary hospitals J2 and J5 and general hospitals K1 and K3 have been selected for emergency treatment. Each directional dashed line connecting disaster areas to temporary hospitals and temporary hospitals to general hospitals is annotated with three values in parentheses. These values represent the number of serious casualties transported, the number of moderate casualties transported, and the number of emergency rescue tools (vehicles or helicopters) deployed. The layout presented in the figure demonstrates a strategy where casualties are transported over short distances to ensure prompt medical intervention, aligning with the principle of efficient rescue operations.

### 5.3 Solution robustness analysis

In this section, several numerical experiments are conducted on the uncertain model to investigate how uncertain parameters influence the objective function.

Given the significant uncertainty in casualty numbers in disaster areas, the study introduces the uncertainty budget parameter $\Gamma_{i}$, which can take real values within the range [0,1]. $\Gamma_{i}=0$ represents that uncertainty is not considered, and the robust model is equivalent to the deterministic model. $\Gamma_{i}=1$ represents extreme uncertainty, where the model needs to handle the worst-case scenario, and the robust model is equivalent to the absolute robust model. $\Gamma_{i}\in (0,1)$ represents a partial consideration of uncertainty, and decision-makers can balance the constraints’ robustness with the solution’s conservatism. Some studies in emergency management have also adopted similar $\Gamma_{i}$ value ranges [8, 59, 64]. To examine the impact of casualty number uncertainty on the objective function, we analyzed the model using different uncertainty budgets and data variability. Specifically, for each uncertainty budget value taken, sensitivity analysis is performed for the data variability of 5%, 15%, and 20% of the uncertainty parameter and its nominal value, respectively. Since the number of casualties must be an integer, we use the rounding method to determine the actual number of casualties in each disaster area, thereby enhancing the accuracy and readability of the analysis.

**Fig 4:**
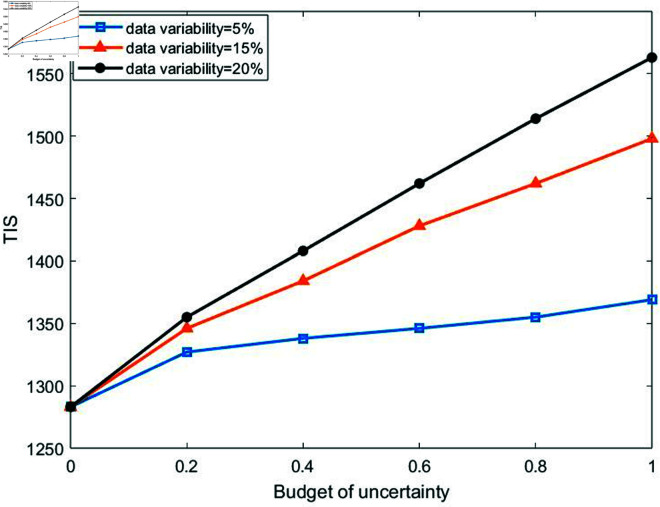
Changes in TIS with respect to uncertainty budget under different data variability.

Fig 4 illustrates the effect of changes in the uncertainty parameters related to the number of casualties on TIS. The results indicate that TIS increases as the uncertainty budget or data variability increases and reaches the worst value when these parameters are at their maximum. This shows that the higher the uncertainty in the number of casualties, the larger the total TIS, and the lower the data variability, the more robust the model.

**Fig 5:**
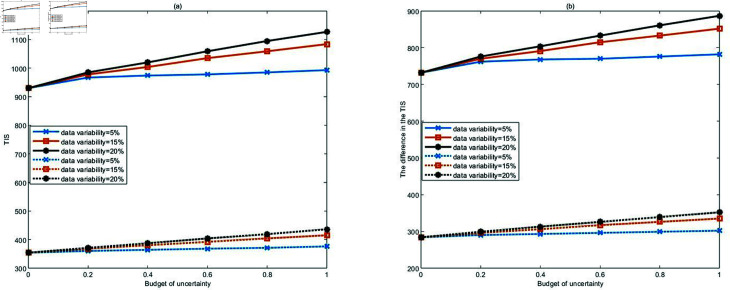
Changes in TIS (a) and TIS difference (b) of different types of casualties with respect to uncertain parameters

Fig 5 illustrates the impact of variations in uncertain parameters on TIS and the TIS difference before and after treatment for different types of casualties. Solid lines represent the TIS trend for serious casualties, while dashed lines represent the trend for moderate casualties as the uncertainty budget increases. In Fig 5a, it can be observed that the TIS for both serious and moderate casualties increases as the uncertainty budget or data variability rises, indicating that a larger uncertainty budget or data variability has a more significant impact on the TIS. This reflects the trade-off between robustness and optimality. Additionally, for the same uncertainty budget and data variability, the TIS for serious casualties is higher than for moderate casualties. Fig 5b illustrates the changes in the TIS difference before and after treatment for serious and moderate casualties concerning uncertain parameters. A larger difference indicates better outcomes after receiving treatment at temporary hospitals. It can be seen from the figure that the treatment outcomes for serious casualties are better after receiving treatment at temporary hospitals. Moreover, as the uncertainty budget increases, the growth rate of the TIS difference before and after treatment slows down, which indicates that the larger the uncertainty budget, the lower the returns in efficiency gains. Fig 5 highlights the following management implications. Managers should allocate medical resources according to injury severity. In particular, when resources such as hospital capacity, rescue vehicles, and helicopters are limited, priority should be given to using these limited resources to treat serious casualties to maximize the effectiveness of the medical rescue operations.

The sensitivity analysis examines the impact of moderate and serious casualties on location decisions and the objective value, as shown in Table 8. The results indicate that although the optimal total TIS varies under different data variability and uncertainty budgets, only three locations are chosen. This further demonstrates the robustness of the model and its insensitivity to parameter changes. Meanwhile, the TIS of casualties significantly decreases after receiving treatment at temporary hospitals compared to before treatment, highlighting the critical role of timely medical interventions in reducing mortality rates and improving treatment outcomes.

**Table 8:** The results in different settings.

Budget of uncertainty	Data Variability (%)	Trauma Index before treatment	Trauma Index after treatment	Objective value	Temporary hospitals Location decisions	General hospitals Location decisions
0	5-20	1150	133	1283	J2, J5	K1, K3
0.2	5	1189	138	1327	J2, J4, J5	K1, K3
	15	1207	139	1346	J2, J4, J5	K1, K3
	20	1215	141	1355	J2, J4, J5	K1, K3
0.4	5	1199	139	1338	J2, J4, J5	K1, K3
	15	1241	144	1384	J2, J4, J5	K1, K3
	20	1262	146	1408	J2, J4, J5	K1, K3
0.6	5	1207	139	1346	J2, J4, J5	K1, K3
	15	1280	148	1428	J2, J4, J5	K1, K3
	20	1311	152	1462	J2, J4, J5	K1, K3
0.8	5	1215	141	1355	J2, J4, J5	K1, K3
	15	1311	152	1462	J2, J4, J5	K1, K3
	20	1357	157	1514	J2, J4, J5	K1,K2,K3
1	5	1227	142	1369	J2, J4, J5	K1, K3
	15	1343	155	1498	J2, J4, J5	K1, K3
	20	1401	162	1563	J2, J4, J5	K1,K2,K3

Some management implications can be obtained from the above results. Before a disaster, policymakers should plan for the establishment of temporary hospitals in high-risk areas, ensuring they are well-equipped with essential medical supplies and staffed with trained personnel. After a disaster, managers should promptly coordinate resources to set up temporary hospitals near affected areas to minimize treatment delays and reduce casualty rates effectively.

**Fig 6:**
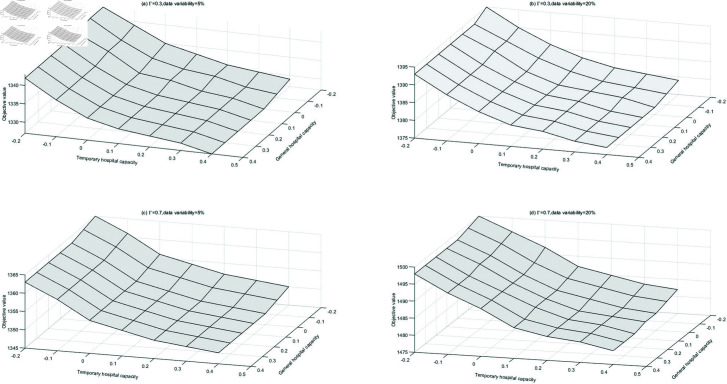
Changes in objective value for increasing hospital capacity.

Fig 6 shows the effect of hospital capacity on the objective function value under the parameters of uncertainty budgets (0.3 and 0.7) and data variability (5% and 20%). The figure shows that the objective function value decreases as the capacity of temporary and general hospitals increases. This indicates that expanding hospital capacity enhances the efficiency of casualty treatment, reducing the overall TIS.

Additionally, a comparison of the slopes of the curves reveals that, under the same uncertainty budget and data variability, the capacity of temporary hospitals significantly impacts the objective function more than that of general hospitals. This can be attributed to the fact that increasing temporary hospital capacity reduces casualty transport time, thereby lowering the TIS. Sun *et al*. [1] suggest that increasing general hospital capacity improves rescue efficiency, which contrasts with our findings, as their study overlooks the crucial role of temporary hospitals in emergency rescue operations. Therefore, in post-disaster emergency response, prioritizing the expansion of temporary hospital capacity is an effective strategy for managers to enhance treatment efficiency and reduce trauma outcomes.

To further validate the robustness of the model, the scale of the test instances was expanded, and comparisons were made between the robust and deterministic models under varying uncertainty budgets and levels of data variability. The number of feasible solutions and their averages were analyzed, as shown in Table 9. The results reveal that the deterministic model fails to provide feasible solutions due to violations of hospital capacity constraints. In contrast, the robust model consistently identifies feasible solutions, regardless of the level of uncertainty. This demonstrates that the robust optimization approach is highly effective in handling uncertainties related to casualty numbers.

**Table 9:** Results of the deterministic and robust models for different problem sizes.

Problem size ∣I∣*∣J∣*∣K∣	Budget of uncertainty	Data Variability (%)	Feasible solutions number		Average of feasible solutions	
			Determine model	Robust model	Determine model	Robust model
9*5*3	0.3	5	8	10	1339.76	1358.81
		15	8	10	1372.23	1454.28
		20	6	10	1393.69	1502.55
	0.7	5	8	10	1358.38	1419.54
		15	5	10	1451.77	1647.54
		20	5	10	1496.81	1756.51
	1	5	8	10	1378.35	1472.27
		15	3	10	1509.24	1791.23
		20	1	10	1496.81	1944.38
12*7*6	0.3	5	8	10	1380.47	1399.81
		15	6	10	1415.18	1489.64
		20	5	10	1436.61	1537.31
	0.7	5	8	10	1399.42	1460.33
		15	0	10	-	1674.20
		20	0	10	-	1778.92
	1	5	6	10	1423.94	1503.60
		15	0	10	-	1807.30
		20	0	10	-	1957.70

## 6 Extension

In the core model analysis, we developed a single-stage robust static location-transportation model to address location problems under uncertain conditions. Although this model provides reasonable location decisions in stable scenarios, its one-time decision structure limits flexibility and reduces effectiveness in adapting to dynamic changes in demand and resource allocation during disaster relief. In real-world situations, factors such as road damage and evolving rescue operations lead to frequent fluctuations in demand and environmental conditions, which highlights the need for greater flexibility in the location of temporary hospitals. Based on this, this section extends the single-stage static model to a two-stage dynamic location model. In the first stage, the model determines the locations of temporary hospitals and initial casualty allocation strategies under uncertain casualty numbers. In the second stage, leveraging the results of the first stage and newly revealed information, the model further optimizes the locations of general hospitals and refines casualty allocation strategies to better adapt to changing conditions.

### 6.1 The formulation of the two-stage dynamic location-transportation model

$$\min F_{1}+F_{2}$$ (27)

$$F_{1}=\sum_{i}\sum_{j}\sum_{s}\sum_{t}p_{ijst}x_{ijst}\omega_{s}$$ (28)

$$F_{2}=\sum_{j}\sum_{k}\sum_{s}\sum_{t}p_{jkst}y_{jkst}\omega_{s}$$ (29)

$$\sum_{i}\sum_{s}x_{ijst}\leq CU_{jt}$$ (30)

$$\sum_{i}\sum_{s}\sum_{t}x_{ijst}\leq C$$ (31)

$$\sum_{i}\sum_{j}\sum_{s}x_{ijst}\leq e^{g}\gamma_{t}$$ (32)

$$\sum_{j}\sum_{t}x_{ijst}=q_{is}$$ (33)

$$\sum_{j}x_{ijst}\leq q_{is}$$ (34)

$$e^{g}\gamma_{t+1}=e^{g}\gamma_{t}-\sum_{i}\sum_{j}\sum_{s}x_{ijst}+\sum_{i}\sum_{j}\sum_{s}x_{ijs(t-1)}$$ (35)

$$\gamma_{1}=\gamma$$ (36)

$$p_{ijst}=p_{s}((t-1)\Delta t+2t_{ij})$$ (37)

$$\sum_{j}y_{jkst}\leq Q_{ks}V_{kt}$$ (38)

$$\sum_{j}\sum_{t}y_{jkst}\leq Q_{ks}$$ (39)

$$\sum_{j}\sum_{s}y_{jkst}\geq V_{kt}$$ (40)

$$\sum_{j}\sum_{k}\sum_{s}y_{jkst}\leq e^{h}\lambda_{t}$$ (41)

$$\sum_{k}y_{jkst}=\sum_{i}x_{ijst}$$ (42)

$$e^{h}\lambda_{t+1}=e^{h}\lambda_{t}-\sum_{j}\sum_{k}\sum_{s}y_{jkst}+\sum_{j}\sum_{k}\sum_{s}y_{jks(t-1)}$$ (43)

$$\lambda_{1}=\lambda$$ (44)

$$p_{jkst}=0.2\cdot p_{s}((t-1)\Delta t+2t_{jk})$$ (45)

$$U_{jt},V_{kt}\in \{0,1\},\forall j\in J,\forall k\in K,\forall t\in T$$ (46)

$$x_{ijst},y_{jkst}\geq 0,\text{and interger}$$ (47)

Here, *T* represents for set of time period , T={t},t=1,2,3,...,n;Δt represents the interval of each time segment {t}.

Similar to Section 4, constraints 33 and 34 can be rewritten as:

$$\sum_{j}\sum_{t}x_{ijst}\geq q_{is}+{\hat{q}}_{is}\Gamma_{i}^{\prime }$$ (48)

$$\sum_{j}x_{ijst}\leq q_{is}-{\hat{q}}_{is}\Gamma_{i}^{\prime }$$ (49)

Thus, the robust counterpart of our proposed model can be reformulated as follows:


$$\min F_{1}+F_{2}$$

s.t. (30)-(32),(35)-(47),(48)-(49).

### 6.2 Solution methodology

Based on the structural characteristics of the model proposed in subsection 6.1, the original problem is decomposed into a master problem (MP) and a subproblem (SP).

The MP is formulated as follows:


$$\left(MP\right)\min\{\sum_{i}\sum_{j}\sum_{s}\sum_{t}p_{ijst}x_{ijst}\omega_{s}+\theta \}$$

subject to constraints (30)-(37), where θ represents the optimal objective function value of the SP.

The SP is formulated as follows:


$$\left(SP\right)\min\{\sum_{j}\sum_{k}\sum_{s}\sum_{t}p_{jkst}y_{jkst}\omega_{s}\}$$

subject to constraints (38)-(45).

The subproblem involves 0-1 binary variables and integer variables, and its non-convex nature prevents the direct application of the duality theory required for dualizing and solving the subproblem in the classical Benders decomposition algorithm. As a result, the classical Benders decomposition struggles to generate effective cutting planes. We employ the Generalized Benders Decomposition (GBD) algorithm to address this limitation. Unlike the classical approach, the GBD algorithm does not depend on the dual form of the SP. Instead, it directly solves the SP in its original form and constructs cutting planes based on the subproblem’s optimal solutions. This information is then fed back to the MP, enabling iterative convergence between the MP and SP. Therefore, the GBD algorithm is employed in this study.

The detailed algorithmic flow is as follows:

**Table:** 

Solution Algorithm Flow	
**Step 1:**	**Initialization** set the upper bound UB=+∞, the lower bound LB=−∞, and the current iteration count *k* = 0.
**Step 2:**	**Solve the master problem** The optimal solution of the MP is obtained as {$x_{k+1}^{*},U_{k+1}^{*},\theta_{k+1}^{*}$}, and the optimal objective function value of the MP is updated to the lower bound LB.
**Step 3:**	**Solve the subproblem** Substitute the solution obtained from the master problem into SP. Obtain the optimal objective function value $F_{2}^{*}$ of the subproblem and the optimal solution {$y_{k+1}^{*},V_{k+1}^{*}$}, and the optimal objective function value of the SP is updated to the upper bound UB.
**Step 4:**	**Iterate until the algorithm terminates** If *UB* − LB≤ϵ, output the optimization result and exit the loop. Otherwise, set *k* = *k* + 1, add the $\theta \geq \sum_{j}\sum_{k}\sum_{s}\sum_{t}p_{jkst}y_{k+1}^{*}\omega_{s}$ to the MP, and return to Step 2.

To evaluate the performance of the GBD algorithm in solving two-stage robust optimization problems, Table 10 presents the test results with different problem sizes. The results indicate that as the problem size increases, the running time increases. However, the problem can still be solved within 2-3 iterations in a relatively short time, and the algorithm consistently produces the optimal solution even under the high uncertainty budget and data variability.

**Table 10:** Algorithm performance under different problem sizes.

Problem size ∣I∣*∣J∣*∣K∣	Budget of uncertainty	Data Variability (%)	Running Time (s)	Iterations (k)	Gap (%)
9*5*3	0.3	5	3.48	2	0
		15	3.53	2	0
		20	3.52	2	0
	0.7	5	3.62	2	0
		15	3.81	2	0
		20	3.65	2	0
	1	5	3.64	2	0
		15	3.66	2	0
		20	3.67	2	0
12*7*6	0.3	5	9.56	2	0
		15	9.73	2	0
		20	10.18	2	0
	0.7	5	10.05	2	0
		15	10.74	2	0
		20	10.88	3	0
	1	5	11.18	2	0
		15	11.19	3	0
		20	11.47	3	0

### 6.3 Calculation results

Table 11 presents the main results of the two-stage dynamic location model under different uncertainty parameters (budget of uncertainty and data variability). Comparing Tables 8 and 11 reveals several key observations: First, as the uncertainty budget and data variability increase, the objective values of both the static and dynamic models show an upward trend. However, the dynamic model’s objective value increases significantly, indicating that it is more sensitive to uncertain conditions. Second, the objective value of the dynamic model is consistently higher than that of the static model across all uncertainty budget and data variability conditions. This demonstrates the dynamic model’s greater flexibility and adaptability in managing uncertainty, though it comes at the cost of higher casualty trauma index values. Finally, regardless of whether the static or dynamic model is used, the location decisions remain consistent under different uncertainty budgets and data variability. This highlights the robustness of the location results, showing that the static model can maintain stable optimal location decisions under various conditions.

**Table 11:** The results in different settings.

Budget of uncertainty	Data Variability (%)	Objective value	Temporary hospitals Location decisions		General hospitals Location decisions	
			*t* = 1	*t* = 2	*t* = 1	*t* = 2
0	5-20	1818	J2, J5	J2	K1, K3	K3
0.2	5	2083	J2, J4, J5	J2, J5	K1, K3	K1, K3
	15	2201	J2, J4, J5	J2, J4, J5	K1, K3	K1, K3
	20	2266	J2, J4, J5	J2, J4, J5	K1, K3	K1, K3
0.4	5	2153	J2, J4, J5	J2, J4, J5	K1, K3	K1, K3
	15	2464	J2, J4, J5	J2, J4, J5	K1, K3	K1, K3
	20	2604	J2, J4, J5	J2, J4, J5	K1, K3	K1, K3
0.6	5	2201	J2, J4, J5	J2, J4, J5	K1, K3	K1, K3
	15	2734	J2, J4, J5	J2, J4, J5	K1, K3	K1, K3
	20	2964	J2, J4, J5	J2, J4, J5	K1, K3	K1, K3
0.8	5	2266	J2, J4, J5	J2, J4, J5	K1, K3	K1, K3
	15	2964	J2, J4, J5	J2, J4, J5	K1, K3	K1, K3
	20	3300	J2, J4, J5	J2, J4, J5	K1, K2, K3	K1, K3
1	5	2370	J2, J4, J5	J2, J4, J5	K1, K3	K1, K3
	15	3204	J2, J4, J5	J2, J4, J5	K1, K3	K1, K3
	20	3615	J2, J4, J5	J2, J4, J5	K1, K2, K3	K1, K3

## 7 Conclusions and future work

This study develops a robust optimization model to optimize the location planning of temporary and general hospitals, casualty transportation, and resource allocation under limited emergency rescue resources under uncertainty of the number of casualties. The rescue chain integrates disaster sites, temporary facilities, and general hospitals and employs the robust optimization method by Bertsimas and Sim [10] to address casualty uncertainties. The model also incorporates dynamic casualty deterioration rates during transportation to minimize the total weighted TIS for two casualty categories. Numerical experiments based on the 2013 Lushan earthquake are conducted to validate the model. Results indicate that changes in serious casualty numbers significantly impact the objective function compared to moderate casualties, and expanding temporary hospital capacity effectively reduces the total TIS. Furthermore, treatment outcomes for serious casualties in temporary hospitals are better than those for moderate casualties. Consequently, prioritizing serious casualties under resource constraints maximizes the efficacy of medical interventions. Finally, the core model is extended into a two-stage dynamic location-allocation framework, revealing that dynamic models enhance flexibility and adaptability in uncertain environments, though with a higher total TIS.

The above findings provide an essential reference for managers of emergency logistics rescue. The following suggestions will help them optimize rescue decision-making and resource allocation. In disaster relief, changes in the number of serious casualties significantly impact rescue efficiency more than moderate casualties. Therefore, managers should prioritize allocating limited medical resources, such as rescue vehicles, helicopters, and hospital capacity, to ensure that serious casualties can receive treatment as soon as possible. In addition, expanding the capacity of temporary hospitals can effectively shorten the treatment time. Therefore, in post-disaster emergency response, managers should prioritize expanding the number and capacity of temporary hospitals. At the same time, the condition of the injured improved significantly after receiving treatment in temporary hospitals. Therefore, managers should quickly organize transportation after a disaster and send the casualties to temporary hospitals for treatment as soon as possible.

This study has several limitations. The model considers only one uncertainty factor, casualty numbers, while real-world disasters involve multiple uncertainties, such as transportation times, rescue costs, and medical resource availability. Future research could incorporate these factors to reflect the complexity of actual disaster scenarios better. Furthermore, the study focuses solely on minimizing the total weighted TIS without addressing other important rescue objectives. Extending the model to a multi-objective robust optimization framework could include goals such as minimizing psychological costs, maximizing survival rates, and reducing response times. Additionally, the robust optimization method used in this study relies on limited information, which may lead to overly conservative solutions. Distributionally robust optimization, which considers probabilistic distribution characteristics, offers a more flexible approach to handling uncertainties and could better balance conservatism and practicality. Future studies could explore this method to improve the precision and adaptability of emergency rescue decision-making.

## Footnotes

1
https://www.sc.chinanews.com.cn/

2
https://www.gov.cn/
